# Clinico-pathological characteristics and treatment outcomes of patients with de-novo metastatic breast cancer: study from a tertiary cancer centre in North-East India

**DOI:** 10.3332/ecancer.2025.1954

**Published:** 2025-07-29

**Authors:** Manas Dubey, Partha Sarathi Roy, Ankur Bhattacharyya, Kakoli Medhi, Rajdeep Bose, Munlima Hazarika, Pompi Daimari Buragohain, Zaveri Mohinta, Anupam Sarma

**Affiliations:** 1 Department of Medical Oncology, BBCI, Guwahati 781016, India; 2 Department of Surgical Oncology, BBCI, Guwahati 781016, India; 3 Department of Onco-Pathology, BBCI, Guwahati 781016, India

**Keywords:** MBC, de novo, Her2, North East

## Abstract

**Background:**

Due to fundamental biological differences, de-novo metastatic breast cancer (MBC) generally exhibits a more favourable prognosis compared to recurrent MBC. There is a notable absence of databases documenting de-novo MBC patients from North-East India.

**Materials and methods:**

A retrospective analysis of 195 patients was performed from 1 January 2020 to 31 December 2022, covering a span of 3 years. Clinical, pathological and radiological data were extracted from medical records.

**Results:**

The median age at diagnosis was 50 years. The median duration of symptoms was 5 months, with 66% of patients being postmenopausal. The predominant histological type was infiltrating ductal carcinoma. Baseline receptor status indicated that 108 patients (55.38%) were hormone receptor (HR) positive, 97 patients (49%) were positive for human epidermal growth factor receptor and 34 patients (16.4%) had triple-negative breast cancer. The most frequent sites of metastasis included bone (28.7%), lung (27%) and liver (17.4%), followed by non-regional lymph nodes (11.8%) and brain (5.6%). Among the 195 patients, 136 (70%) received treatment. Seventy-three patients (37.4%) underwent single-agent chemotherapy with taxanes, 48 patients (24.2%) received poly-chemotherapy and 12 patients (6.15%) were treated with up-front hormone therapy. Of the 110 patients who were HR positive, 57.2% received endocrine therapy (15 patients (13.6%) on tamoxifen and 48 patients (43.6%) on aromatase inhibitors). Among the 97 patients who were Her2-neu positive, 63 patients (65%) received trastuzumab-based therapy. The study reported a 3-year overall survival rate of 24%. Multivariate analysis indicated that the presence of oligo-metastasis, along with Her2-positive and HR-positive status, correlated with improved patient outcomes.

**In conclusion**, our findings suggest that patients with Her2-positive, HR-positive and oligometastatic disease experience significantly enhanced outcomes. Enhancing access to novel therapeutic options for our patient population is likely to result in improved prognoses.

## Introduction

Breast cancer represents the most prevalent form of cancer among women in India, constituting 28.2% of all female cancer cases [1, 2]. Since 1990, there has been an approximate 40% rise in the age-standardised incidence rate of breast cancer within the country [3]. According to the Indian Council of Medical Research, this malignancy is particularly common among females in North-East India [4]. The heterogeneous nature of breast cancer biology results in approximately 5% to 8% of newly diagnosed patients presenting with distant metastases at the time of initial diagnosis [5]. The most frequently affected metastatic sites include the bones, lungs and liver [6, 7]. Additionally, around 20% of patients experience brain metastases, which significantly impacts survival rates [8]. In India, the incidence of metastatic breast cancer (MBC) ranges from 5% to 25% [9, 10]. Studies conducted in India indicate that MBC is associated with poor prognoses, reflected in a 5-year survival rate of only 18% to 22% [11–13]. Contributing factors to these unfavourable outcomes include younger age at diagnosis, advanced disease stage at presentation, delays in treatment initiation and limited access to modern therapeutic options [14]. The primary treatment approach for MBC involves systemic therapy, which encompasses chemotherapy, endocrine therapy, immunotherapy and targeted therapies [15]. The selection of optimal treatment strategies is contingent upon the characterisation of MBC, taking into account factors such as age, menopausal status, the number and locations of metastases, histological type and crucially, molecular characteristics including hormonal receptor estrogen receptor/progesterone receptor (ER/PR) status and Her2 expression [16, 17]. There is a notable absence of databases documenting de novo MBC patients from North-East India. Consequently, this study aims to investigate the prognostic factors and treatment outcomes for MBC with de novo metastasis at Dr. B. Borooah Cancer Institute in Guwahati, Assam, which serves as the largest tertiary cancer care facility in the state.

## Materials and methods

### Study design and ethical considerations

This investigation is a retrospective analysis of patients diagnosed with de-novo MBC who attended the breast clinic at Dr. B. Borooah Cancer Institute. The study focused on patients presenting with metastatic disease at initial diagnosis from January 2020 to December 2022, covering a 3-year period. Data concerning baseline clinical characteristics, pathological findings and treatments administered were extracted from our computerised database and clinical case files maintained by the medical records department. Patients with incomplete data, specifically those lacking information on metastasis, Her2/neu status or hormone receptor (HR) status, were excluded from the analysis. Approval for the study was obtained from the ethics committee of the Institute. HR status and Her2/neu were assessed using standard immunohistochemical (IHC) techniques. Nuclear staining of more than 1% of tumour cells was deemed positive for ER and PR [18]. Patients were classified as Her2 positive if they exhibited IHC 3+ positivity or if fluorescence *in situ* hybridisation (FISH) indicated amplification. The testing for Her2/neu status adhered to the guidelines established by ASCO and the College of American Pathologists [19]. Upon presentation, all patients underwent a comprehensive evaluation that included a detailed clinical history, physical examination and various radiological assessments (bone scintigraphy, computed tomography, magnetic resonance imaging and positron emission tomography). At our institute, the treatment protocols for MBC encompass chemotherapy (either single-agent or combination therapy), hormone therapy (HT) utilising tamoxifen or aromatase inhibitors and targeted therapies such as trastuzumab or CDK4/6 inhibitors (palbociclib or ribociclib) in conjunction with HT, contingent upon receptor status. The specific chemotherapy regimens are detailed in Table 1. Statistical Analysis Nominal data were expressed as counts and percentages, while continuous data were represented as median values with ranges. The median follow-up duration was determined using the reverse Kaplan–Meier method. Overall survival (OS) was defined as the time from the initiation of treatment to the last follow-up visit or the occurrence of death. Those patients who completed at least 3 cycles of chemotherapy were taken up for survival analysis. The analysis was conducted using SPSS Version 21.0 (IBM Corp., Armonk, NY).

## Results

**Figure 1.**
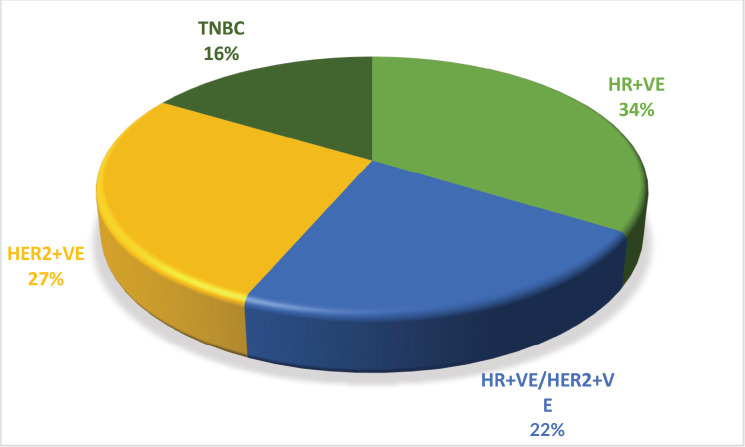
Receptor status of MBC patients. HR = Hormone receptor, Her2 = Human epidermal growth factor receptor2/neu, TNBC = Triple negative breast cancer.

**Figure 2.**
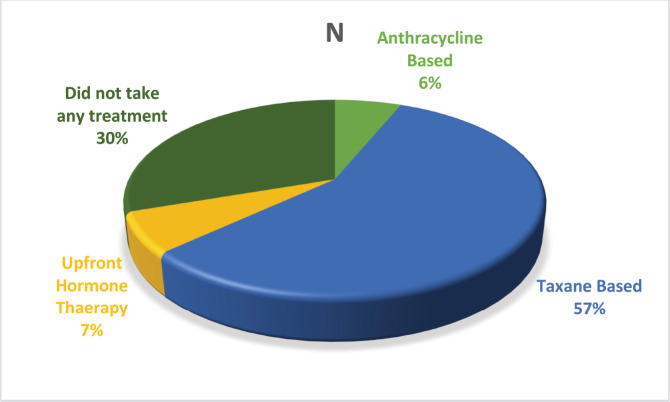
Treatment administered to the patients.

**Figure 3.**
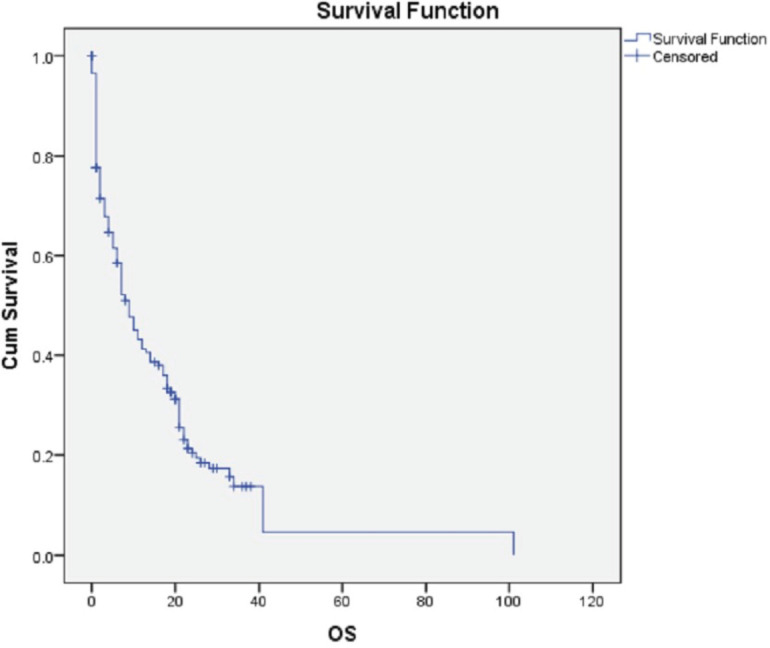
Kaplan Meir curve of OS (in months).

Over the course of the 3-year study, a total of 2,417 breast cancer patients were registered within the breast unit, of which 240 patients (10.0%) were identified with de novo metastasis. A total of 45 patients were excluded from the analysis due to the absence of baseline clinical details or information regarding ER, PR and Her2/neu status, including FISH for IHC 2+. Consequently, 195 patients (8.1%) were included in the final analysis. The baseline characteristics and treatment regimens are summarised in Table 2. The median age at diagnosis was 50 years, with a range from 21 to 83 years. The median duration of presenting symptoms was 5 months. Notably, 66% of the patients were post-menopausal. The distribution of breast cancer laterality was as follows: right-sided in 91 patients (47%), left-sided in 96 patients (49.2%) and synchronous bilateral in 8 patients (4.1%). Metastatic involvement was categorised as a single organ in 47%, two organs in 30.7% and more than two organs in 22%. The most frequently observed sites of metastasis included bone (28.7%), lung (27%), liver (17.4%), both liver and lung (9.7%), non-regional lymph nodes (11.8%) and brain (5.6%). The predominant histological type was infiltrating ductal carcinoma (IDC), accounting for 187 patients (95.8%), followed by infiltrating lobular carcinoma in 6 patients (3%). HR positivity (ER, PR or both) was noted in 66 patients (33.8%), while both HRs and Her2-neu were positive in 44 patients (22.5%) (Figure 1). Her2-neu positivity alone was observed in 53 patients (27%) and 34 patients (16.4%) were classified as triple-negative. Approximately 114 patients (58.4%) exhibited oligometastatic disease, defined as five or fewer metastases involving one or two organs, while the remaining patients did not fulfill the criteria for oligometastasis. Among the 195 patients, 136 (69.7%) underwent treatment. Of these, 73 patients (37.4%) received single-agent chemotherapy with taxanes (either paclitaxel or docetaxel), and 48 (43.6%) aromatase inhibitor) (Figure 2). Among the 96 patients identified as Her2-neu positive, 63 patients (65.6%) were treated with trastuzumab-based therapy. Additionally, 14 patients (9.4%) received CDK4/6 inhibitors, specifically palbociclib or ribociclib, in conjunction with HT. The median follow-up period for this study was 29 months, during which the median OS for the entire cohort was recorded at 14 months, as illustrated in Figure 3. The 3-year OS rate was found to be 24%. Multivariate analysis indicated that factors such as oligo-metastasis, Her2 positivity and HR positivity were significantly correlated with improved outcomes.

## Discussion

Breast cancer represents the most prevalent malignancy among women in India [1]. The northeastern region of the country exhibits a notably high incidence of this disease. Approximately 82% of the population in this area resides in rural settings, with a significant portion being from tribal communities. Survival rates for cancer patients in these states are comparatively lower, with a greater number of cases diagnosed at an advanced stage characterised by distant metastasis [4]. To date, there has been no comprehensive study conducted in North-East India to assess the clinico-pathological characteristics of patients with de-novo MBC. This study aims to document various factors, including age, menopausal status, HR status, Her2 status, histological type, the location and number of distant metastases and the type of treatment administered. To the best of our knowledge, this represents the largest study of its kind from the northeastern region of India. According to Western literature, 5%–8% of breast cancer patients present with distant metastases at diagnosis, while Indian studies indicate an incidence ranging from 6% to 25% [20]. In our study, 8.1% of patients exhibited metastasis at the time of presentation. A separate Indian study reported that 22% of patients presented with upfront metastasis, a figure significantly higher than that observed in our cohort [21]. The median age of participants in our study was 50 years, which is nearly a decade younger than that reported in Western breast cancer populations [14]. Data from various hospital-based cancer registries indicate that 80% of Indian breast cancer patients are under the age of 60. Registries from Dibrugarh, Delhi, Jaipur and South India have reported average ages of 44.2, 46.8, 47 and 49.6 years, respectively. Similarly, population-based studies have shown that the mean age of breast cancer patients falls between 50 and 53 years [10]. Gogia *et al* [21] found a median age of 49 years (range 26–73), which aligns with our findings. In contrast, Dafni *et al* [23] reported a median age of 60 years, while another study involving 716 breast cancer patients by Weide *et al* [24] noted a median age of 61 years [21–24].

Our investigation revealed that approximately 66.7% of the participants were postmenopausal. An analysis encompassing six consecutive clinical trials conducted over a span of 20 years, which included 640 patients with MBC, indicated that 71% of individuals in the postmenopausal cohort were postmenopausal [22]. Additionally, a retrospective study from Germany involving 716 MBC patients found that 80% were postmenopausal. Comparable proportions have been reported in other studies concerning MBC [21–25]. In our cohort, IDC was the predominant histological type, accounting for 95.6% of cases, followed by invasive lobular carcinoma. A SEER analysis focusing on Asian Indian women identified a higher prevalence of IDC (69.1% compared to 65.6%) and a lower incidence of invasive lobular carcinoma (4.2% versus 8.2%) when compared to a Western patient population [26]. Furthermore, a study conducted in Northeast India at a regional cancer center found that IDC was the most frequently observed histology in both triple-negative breast cancer and other subtypes [27]. We have compiled a comparative analysis of our current findings with previously published literature, as illustrated in Table 3. The most common sites of distant metastasis in breast cancer are the bones, followed by the lungs, liver, non-regional lymph nodes and brain [7]. This series reflects similar metastatic patterns. Previous studies have reported HR status among de novo MBC patients ranging from 49% to 79% [20–24]. In our study, HR positivity and Her2-neu positivity were observed in 56.3% and 49% of patients, respectively. Notably, our findings indicate a higher rate of Her2-neu overexpression compared to earlier studies, underscoring the distinct biological characteristics of breast cancer in patients from Northeast India [20, 27].

In this investigation, approximately 62% of the participants underwent chemotherapy, either as a single agent or in combination with other agents, while 57.2% received HT following chemotherapy. Among the 97 patients who tested positive for Her2/neu, 63% were treated with trastuzumab. A single-center study conducted in India involving 500 patients with MBC revealed that only 50% of patients could access trastuzumab due to financial limitations, which is significantly lower than the findings of our study [28]. The timeframe of our research spanned from 2020 to 2022, during which the COVID-19 pandemic severely impacted healthcare services globally, affecting breast cancer patients as well. Approximately 70% of these patients were unable to obtain life-saving treatments [29, 30]. This situation may contribute to the limited access to therapy observed in our study, alongside other factors such as lack of awareness, delayed diagnosis, insufficient access to healthcare professionals and inadequate funding for treatment [14]. The current study identified a greater proportion of patients presenting with oligo-metastatic breast cancer (OMBC) compared to other research [27, 28]. Although the exact incidence of OMBC remains poorly defined, existing data indicate that a notable segment of de-novo MBC cases presents as oligo-metastatic disease [31]. The standard treatment approach for OMBC continues to be a subject of debate. Research examining the impact of surgical resection of the primary tumour in conjunction with systemic therapy for OMBC has yielded inconsistent outcomes [27, 32, 33]. Several limitations of this analysis warrant attention. First, the study is confined to a single center with a relatively small patient cohort. Second, our database does not provide comprehensive information regarding chemotherapy, HT and anti-Her2 therapy. Third, data on co-morbidities and performance status were not included in our database. Finally, the median follow-up duration for our cohort was relatively brief (29 months).

**In conclusion**, to the best of our knowledge, this represents the largest study of de-novo MBC conducted in North-East India. Our findings indicate that Her2-positive, HR-positive and oligometastatic disease are associated with significantly improved outcomes. Additionally, our cohort is characterised by a younger demographic.

## List of abbreviations

IDC, Infiltrating ductal carcinoma; ILC, Invasive lobular carcinoma; Her2, Human epidermal growth factor receptor2/neu; HR, Hormone receptor.

## Conflicts of interest

Nil.

## Funding

Nil.

## Figures and Tables

**Table 1.** Chemotherapy regimens.

Regimens	
Multi-agent	Doxorubicin (A = 60 mg/m^2^) / Cyclophosphamide (C = 600 mg/m^2^) followed by Paclitaxel (175 mg/m^2^) or Docetaxel (75 mg/m^2^) (4 cycles of AC f/b 4 cycles of Taxane, every 3 weekly)
	Docetaxel (T = 75 mg/m^2^) / Cyclophosphamide (C = 600 mg/m^2^) 6 cycles of TC every 3 weekly
	TCH regimen (H = trastuzumab (8 mg/kg loading followed by 6 mg/kg), Cb = carboplatin (AUC = 6), and T = docetaxel (75 mg/m²) every 3 weekly for 6 cycles).
Single agent (SA)	Single agent Taxanes either with Paclitaxel (175 mg/m^2^) or Docetaxel (75 mg/m^2^) every 3 weekly for 4–6 cycles

**Table 2.** Baseline characteristics. (Continued)

Characteristics		No. of patients (%)
Total no. of patients		195 (8.1)
Median age in years (Range)		50 (21–83)
Median duration of symptoms		5 Months
Gender: Female		195 (100)
Menopausal status	Premenopausal	65 (33.3)
	Post-menopausal	130 (66.7)
Side	Left	96 (49.2)
	Right	91 (47)
	Bilateral	8 (4.1)
Histopathology at baseline	IDC	187 (95.8)
	ILC	6 (3)
	Others	2 (1)
Receptor status	HR positive	66 (33.8)
	HR and Her2 neu positive	44 (22)
	Her2 neu positive	53 (27)
Oligometastatic disease (less than or equal to 5 metastases)	114 (58.4)	32 (16.4)
Site of metastases	Bone only	56 (28.7)
	Lung	52 (27)
	liver	34 (17.4)
	Liver plus lung	19 (9.7)
	Non regional LN	23 (11.8)
	Brain	11 (5.6)
Therapy (*n* (%)) = 136 (69.7)	Single-agent chemotherapy (Taxanes)	73 (37.4)
	Multi-agent chemotherapy	48 (24.6)
	Others	3 (1.5)
	Upfront HT	12 (6.15)
	Tamoxifen	15 (13.6)
	Aromatase inhibitor	48 (43.6)
	Trastuzumab	63 (65)

IDC = Infiltrating ductal carcinoma, ILC = Invasive lobular carcinoma, HR = Hormone receptor, Her2 = Human epidermal growth factor receptor2/neu

**Table 3.** Comparison of our study with previous published studies.

Parameters	Dafni et al [23]	Weide et al [24]	Giordano et al [25]	Gennari et al [22]	Gogia et al [21]	Current study
No.	364	716	105	174	375	195
Study period	2003–2006	1995–2013	1995–2000	1998–2001	2012–2018	2020–2022
Median age in years, (Range)	60 (27–84)	61	49 (26–73)	<50:35%	49 (26–73)	50 (21–83)
				50–70:36%		
				>70: 28.7%		
Premenopausal status	19.5	19	48	28.7	40	33.3
Post-menopausal status	80	80	52	71	60	66.7
HR positive	68	79	49	56.9	62.4	56%
Her 2/neu positive	-	20	-	-	38.6	49%
Bone only metastases	50	36	28	58.5	26.7	28.7
Visceral site metastases	72	47	37	41.5	58.4	54.1
Non-regional lymph nodes	25	9	30		5.6	11.8
Brain metastases	-	4	-		2.7	5.6

HER2, human epidermal growth receptor 2
